# Prevalence, distribution and severity of tongue coatings according to the proposed classification of tongue coatings severity index: a gender-based evaluation

**DOI:** 10.1186/s12903-025-07139-z

**Published:** 2025-11-12

**Authors:** Rajashri Abhay Kolte, Abhay Pandurang Kolte, Vinisha Bajaj, Ritika Rakesh Gattani, Pavan Bajaj, Shivani Thakre, Mahima Kothekar

**Affiliations:** 1 Ranjeet Deshmukh Dental College and Research Centre, Nagpur, India; 2 Sharad Pawar Dental College and Hospital, Datta Meghe Institute of Higher Education and Research, Sawangi (Meghe), Wardha, India

**Keywords:** Tongue coating, Oral malodour, Gingivitis, Periodontitis, Tongue coating index

## Abstract

**Objectives:**

Existing methods to calibrate tongue coatings on the dorsum of the tongue have limitations of subjectivity, complexity, or time inefficiency. The proposed Tongue Coatings Severity Index (TCSI) was designed to fill this gap by offering a simple, reproducible, and time-efficient method for assessment. This study investigated the prevalence, distribution, and severity of tongue coatings and their clinical associations.

**Materials and methods:**

A cohort of 200 systemically healthy patients (20–60 years) with equal gender distribution were screened for Tongue Coating Area (TCA), Tongue Coating Severity (TCS), Final Tongue Coating Severity (FTCS), and oral malodour, and compared according to periodontal status.

**Results:**

The mean TCA was higher in males, whereas TCS and FTCS showed higher values in females, although these differences did not achieve statistical significance. TCA, TCS, and FTCS scores increased with worsening periodontal status and were significantly higher in patients with oral malodour.

**Conclusion:**

The TCSI enables clinicians to record both the extent and severity of tongue coatings quickly and reliably, facilitating its use in screening halitosis, monitoring periodontal disease progression, and potentially in public health surveillance. Validation in larger and more diverse populations is required prior to widespread adoption.

**Trial registration:**

Clinical Trials Registry (CTRI/2022/02/040688) last modified on 26/02/2022 and registered on 28/02/2022.

## Introduction

The dorsum of the tongue comprises of papillary structure which is a distinct ecological niche [1, 2]. Microorganisms of the tongue may contribute to dental plaque accumulation within the oral cavity [3]. There is constant shedding of superficial epithelium of tongue, Sarrazin depicted that dorsum of the tongue is barely ever free of microbes which in some of the cases can contribute up to 90% of the bacterial mass as it acts as one of the niches for microbial adherence [4]. It has been proposed that bacteria from the tongue, particularly its posterior region, can colonize the tonsils, teeth, and gingiva. The microbes present within the coatings are supposed to be closely associated with dental plaque and periodontal disease. Daily tongue cleaning is thus recommended, with the optimal time being in the morning usually on an empty stomach, as this may trigger gagging or even vomiting. Tongue hygiene has been practiced routinely for centuries by most of the eastern cultures [5]. Despite its importance, tongue hygiene has received relatively little attention in recent decades. In traditional medicine, examining the tongue has long been regarded as a vital tool for diagnosis and assessing prognosis—a practice that dates back to 16th century BC to bone inscriptions and primitive tortoise shell. Methods of traditional inspection of tongue have been published in many articles and books on tongue coating [6, 7]. But with ignorance towards this aspect of oral hygiene, it has become cumbersome to grasp the true value of this method.

Dorsum of tongue presents tremendous variability in terms of its appearance. It may either be reddish pink or a thin white coating [7], and the older adults are prone to tongue discoloration due to dietary changes, reduced salivary flow, and challenges in maintaining proper oral hygiene. The thickness of coatings on the tongue varies depending upon the number of microorganisms and other debris embedded in it. Patients having periodontal disease are more prone to have a thick layer of coating compared to patients having periodontal conditions in good health [8, 9]. The dorsum of the tongue provides an extensive surface area that promotes the accumulation of oral debris and microbial colonization, with the coating comprising bacteria, desquamated epithelial cells, leukocytes from periodontal pockets, blood-related metabolites, and various nutrients [10]. 

A number of methods have been described to measure the extent and severity of coatings on the tongue. Yaegaki and Sanada [8] suggested a useful method to measure the tongue coating which involved removing of the coating with a small spoon type tongue scraper, from the terminal sulcus to the apex of the tongue. This was followed up with cleaning of the tongue dorsal surface with the use of small cotton pellets immersed in saline. Following the removal of tongue coating, its wet weight was measured in milligrams. Gross et al. introduced an index ranging from 0 to 3 (indicating no coating to severe coating); however, they did not provide clinical descriptions or photographic references to illustrate the index [11]. These calibrations appeared arbitrary, lacked clinical relevance, and were difficult for clinicians to interpret. Bosy et al. visually assessed the amount of coating on the dorsal surface of the tongue, categorizing it as heavy, moderate, light, or absent [2]. This method again was privy to subjective bias. Miyazaki et al. evaluated tongue coating based on its coverage area on the tongue dorsum, assigning scores as follows: 0 for no visible coating, 1 when less than one-third of the surface was covered, 2 when less than two-thirds was coated, and 3 when more than two-thirds was covered [12]. Chen categorized tongue coating based on its color—white, yellow, grey, or black—and the quality of the tongue surface, which included characteristics such as dry, slippery, dry and rough, prickly, partially furred, and completely furred [7]. However, these methods seem to be complicated and confusing with a lot of subjectivity associated with the evaluation.

Furthermore, the oral cavity is widely regarded as a mirror of systemic health, with mucosal and microbial changes often reflecting underlying systemic conditions. Studies have reported that systemic viral infections, gastrointestinal disturbances, and respiratory diseases may manifest as tongue changes, reinforcing the clinical relevance of standardized tongue coating assessment. Expanding the scope of tongue coating evaluation to such systemic associations enhances its clinical value beyond periodontal health alone [13]. 

Considering the limitations of existing indices, there remains a need for a method that is both clinically simple and reproducible, while also minimizing subjectivity. The proposed Tongue Coatings Severity Index (TCSI) was designed with these attributes in mind, offering a practical tool that can be applied efficiently in both research and clinical practice. Recent investigations have demonstrated that tongue coating is not only associated with oral malodour but also reflects distinct microbial and metabolic characteristics, further underscoring the importance of a standardized and clinically feasible index for its assessment [14]. Therefore, the present study was undertaken to evaluate the prevalence, distribution, and severity of tongue coatings using the proposed classification, with a focus on its clinical applicability.

## Study population and methodology

The study design and protocol were thoroughly explained to the patients, and informed consent was obtained from each participant before their enrolment in the study. A total of 200 patients, equally divided into two groups based on gender and aged between 20 and 60 years, were enrolled in the study according to the following inclusion criteria.: (a) systemically healthy patients (b) patients with no evidence of anatomical anomaly or pathology. The patients were excluded in cases of: (a) any medications (b) presence of any overt dental infection or dental caries d) with a history of any surgical therapy within the past six months and e) current smokers. The included patients were then diagnosed according to their periodontal condition as periodontally healthy, gingivitis and periodontitis based on the current classification of periodontal diseases [15]. 

The selected patients were screened for the prevalence, distribution and severity of tongue coatings according to the proposed classification of Tongue Coatings Severity Index as follows.

### Proposed tongue coating severity index

**Fig. 1:**
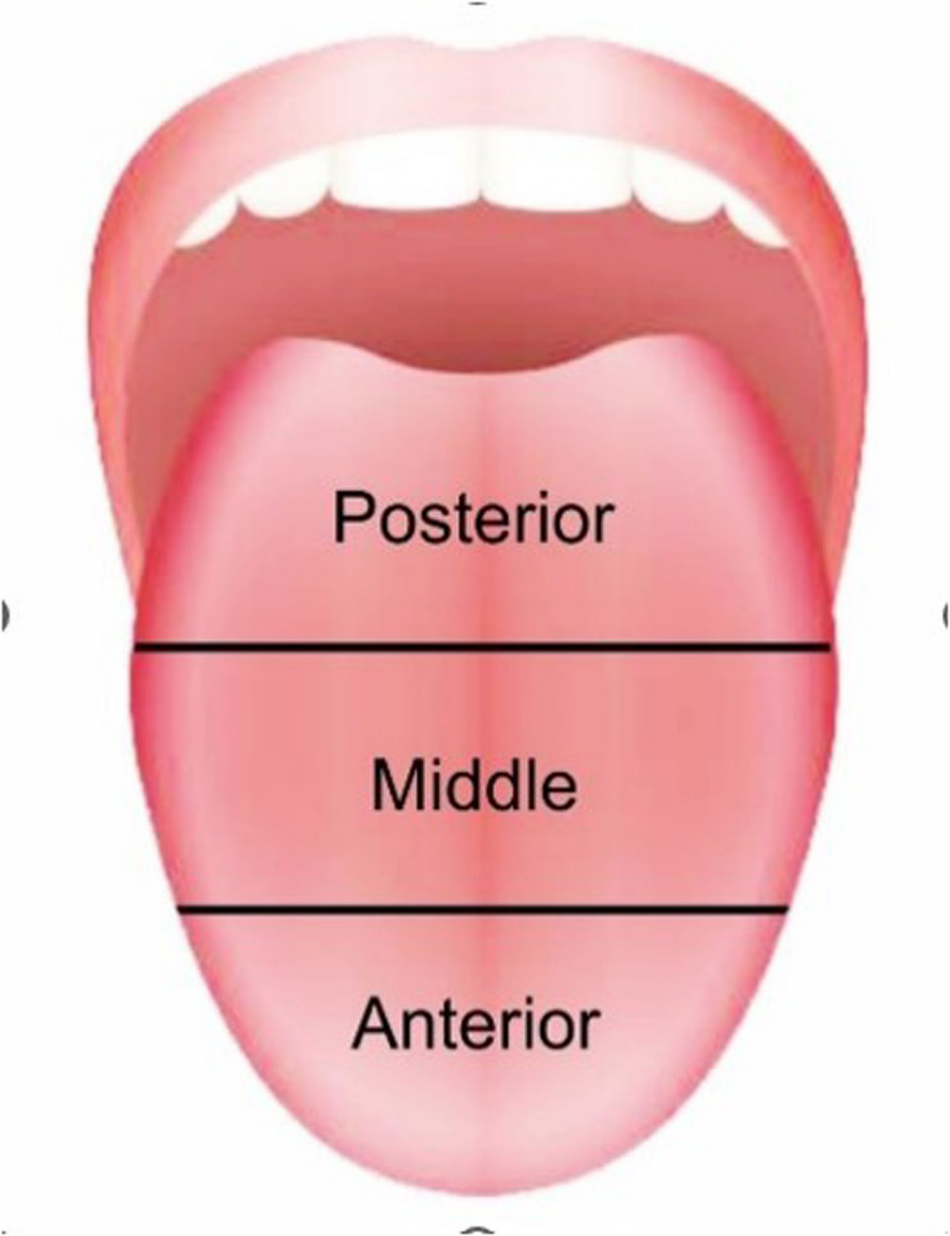
Division of the tongue antero-posteriorly

According to the index, dorsum of the tongue is divided into three equal parts antero-posteriorly (Fig. 1). The surface coatings present in each of these parts are calculated separately and then the scores of all the three parts are added. The scoring criteria for each of these parts is given as:

#### A1) anterior tongue coating area score (Fig. 2a)

**Fig. 2:**
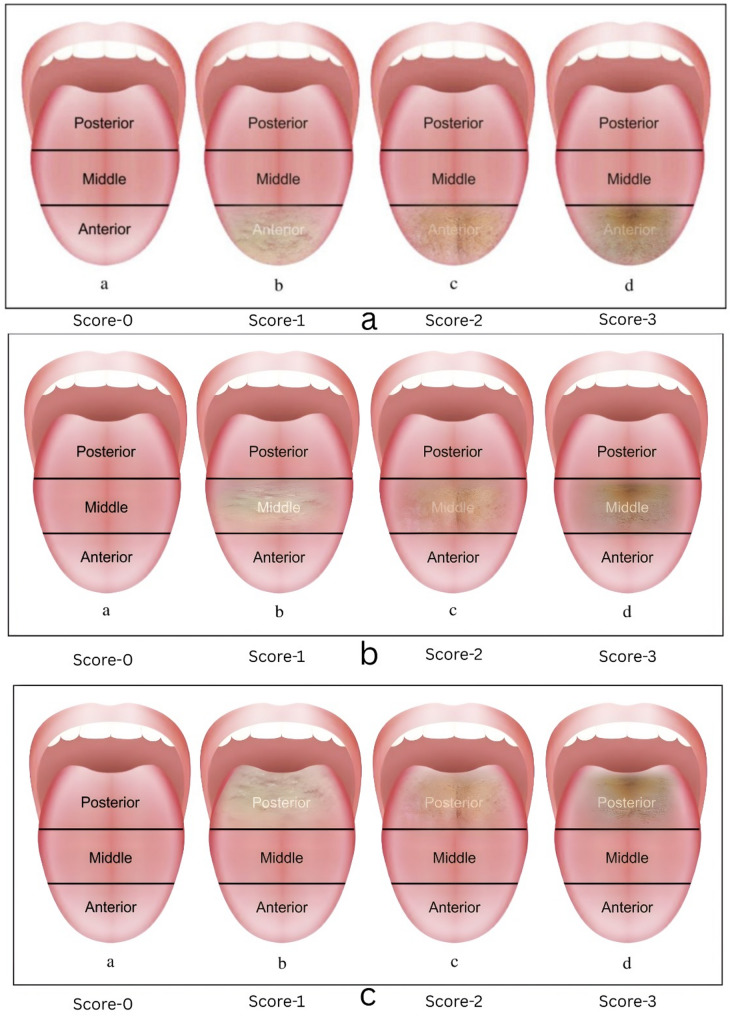
**a:** Anterior tongue coating area score **b**: Middle tongue coating area score **c**: Posterior tongue coating area score

- 0 – No surface coating visible on the entire portion of anterior part of tongue.
- 1 – Surface coating visible in less than one third of anterior part of the tongue.
- 2 - Surface coating visible in more than one third but less than two third of the anterior part of tongue.
- 3 - Surface coating visible in more than two third of the anterior part of the tongue.

#### A2) middle tongue coating area score (Fig. 2b)

- 0 – No surface coating visible on the entire portion of the middle part of tongue.
- 1 – Surface coating visible in less than one third of the middle part of tongue.
- 2 - Surface coating visible in more than one third but less than two third of the middle part of the tongue.
- 3 - Surface coating visible in more than two third of the middle part of the tongue.

#### A3) posterior tongue coating area score (Fig. 2c)

- 0 – No surface coating visible on the entire portion of the posterior part of tongue.
- 1 – Surface coating visible in less than one third of the posterior part of tongue.
- 2 - Surface coating visible in more than one third but less than two third of the posterior part of the tongue.
- 3 - Surface coating visible in more than two third of the posterior part of the tongue.

The Total tongue coating area score is calculated by addition of the above three area scores.

So, according to the above criteria.

- a) Maximum Total Tongue Coating Area Score will be: 9.
- b) Minimum Total Tongue Coating Area Score will be: 0.

$$\begin{aligned}\mathbf{Total}&\boldsymbol\;\mathbf{Tongue}\boldsymbol\;\mathbf{Coating}\boldsymbol\;\mathbf{Area}\boldsymbol\;\mathbf{Score}\;\;=\\&\;\mathrm{Anterior}\;\mathrm{tongue}\;\mathrm{coating}\;\mathrm{area}\;\mathrm{score}\;+\\&\;\mathrm{Middle}\;\mathrm{tongue}\;\mathrm{coating}\;\mathrm{area}\;\mathrm{score}\;+\\&\;\mathrm{Posterior}\;\mathrm{tongue}\;\mathrm{coating}\;\mathrm{area}\;\mathrm{score}\end{aligned}$$

The severity of the coating is calculated by colour of the coating on each of these areas as Tongue coating severity score.

#### B1) anterior area tongue coating severity score (Fig. 3a)

**Fig. 3:**
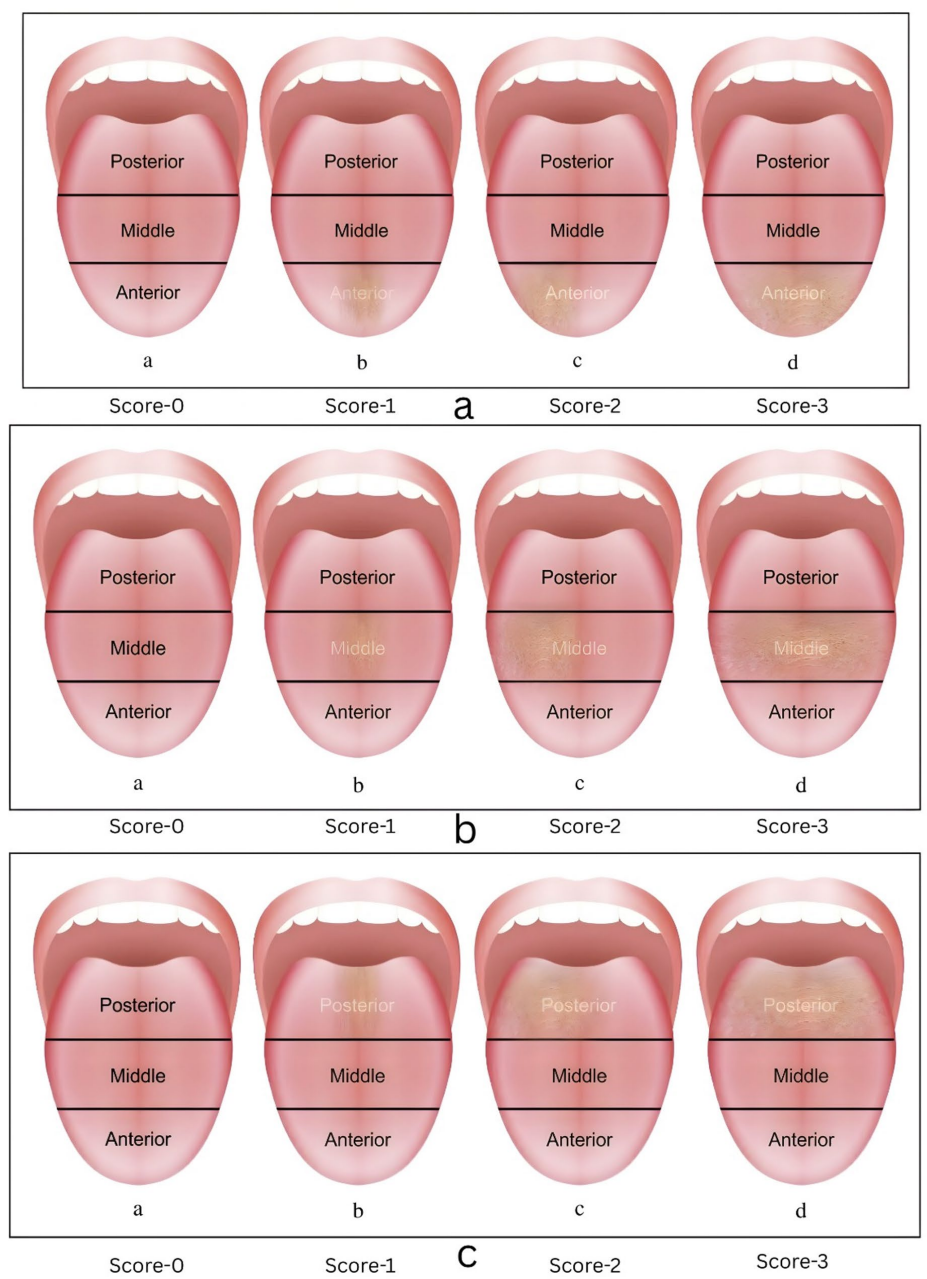
**a**: Anterior area tongue coating severity score **b**: Middle area tongue coating severity score **c**: Posterior area tongue coating severity score

- 0 – No surface coating visible on the entire portion of the anterior part of tongue.
- 1 – White surface coating visible in anterior part of tongue.
- 2 – Yellowish white surface coating visible in anterior part of tongue.
- 3 – Yellow brown surface coating visible in anterior part of tongue.

#### B2) middle area tongue coating severity score (Fig. 3b)

- 0 – No surface coating visible on the entire portion of middle part of tongue.
- 1 – White surface coating visible in middle part of tongue.
- 2 – Yellowish white surface coating visible in middle part of tongue.
- 3 – Yellow brown surface coating visible in middle part of tongue.

#### B3) posterior area tongue coating severity score (Fig. 3c)

- 0 – No surface coating visible on entire portion of the posterior part of tongue.
- 1 – White surface coating visible in posterior part of tongue.
- 2 – Yellowish white surface coating visible in the posterior part of tongue.
- 3 – Yellow brown surface coating visible in the posterior part of tongue.

So, according to the above criteria.

- c) Maximum Total Tongue Coating Severity Score will be: 9.
- d) Minimum Total Tongue Coating Severity Score will be: 0

The total tongue coating severity score is calculated by addition of the above three scores. The final tongue coating severity score is calculated as.

$$\begin{aligned}\mathbf{Final}&\boldsymbol\;\mathbf{Tongue}\boldsymbol\;\mathbf{Coating}\boldsymbol\;\mathbf{Severity}\boldsymbol\;\mathbf{Score}\;=\\&\;\mathrm{Total}\;\mathrm{Tongue}\;\mathrm{Coating}\;\mathrm{Area}\;\mathrm{Score}\;+\\&\;\mathrm{Total}\;\mathrm{Tongue}\;\mathrm{Area}\;\mathrm{Severity}\;\mathrm{Score}\end{aligned}$$

So, according to the above criteria.

- e) Maximum Final Tongue Coating Severity Score will be: 18.
- f) Minimum Final Tongue Coating Severity Score will be: 0.

Based on the Final Tongue Coating Severity Score the tongue coatings will be labelled as:

$$\begin{aligned}&\mathbf{Mild}\;-\;0\;\mathrm{to}\;6,\;\mathbf{Moderate}\;-\;6.1\;\mathrm{to}\;12,\\&\;\mathbf{Severe}\;-\;12.1\;\mathrm{and}\;\mathrm{above}\end{aligned}$$

### Consistency of observation

Digital photographs of all the patients were obtained from a uniform 2 feet distance so as to avoid impinging on patient’s personal space. These photographic images were observed with 1680 × 1050-pixel resolution with the help of Adobe Photoshop (Adobe Systems). Standardization with 1:1.2 as an enlargement ratio of these photographs was done using the area of interest which was the dorsum of tongue. The camera was placed at 90 degrees to the patient to prevent the illusion of canted or inverse lip line. To obtain consistency, reliability and reproducibility of the proposed criteria, two observers (RK, PT) screened and scored all the patients and the mean of their scores was considered as the final score for each of the patients. Both the examiners were calibrated prior to the study for these recordings and the intraclass correlation for tongue coating scores, coating of the tongue severity and degree of tongue coating was found to be reliable. (Table 1)

**Table 1:** Inter examiner reliability between both examiners

	Tongue coating area score	Tongue coating severity	Degree of tongue coating
Intra class correlation	*r* = 0.871	*r* = 0.912	*r* = 0.941
P value, Significance	*p* < 0.001**	*P* < 0.001**	*P* < 0.001**

Intraclass correlation (*r* = 0.871) for tongue coating area score and *r* = 0.912 for tongue coating severity which was found to of highly statistical significance (*p* < 0.001)

### Statistical analysis

Data entries were done in Microsoft Office Excel 2010 and analyses of results were performed using Statistical product and service solution (SPSS) version 22 software. Descriptive statistics such as mean and standard deviation were calculated for quantitative variables. The p value was fixed at 0.05. Data normality was checked using Shapiro Wilk test. Unpaired t test was used for comparison of tongue coating scores between males and females. One-way Anova f test was used for overall comparison among three study groups based on clinical diagnosis. Tukey’s post hoc test was used for pairwise comparison between groups in relation to tongue coating scores between different clinical diagnosis. Comparison between tongue coating scores with presence and absence of halitosis group was done using unpaired t test. Inter examiner reliability between examiners was done using intra class correlation test for tongue coating parameters.

## Results

A total of 242 patients were screened and 200 were found to be suitable for the trial. Amongst the total study population 75 patients were diagnosed as periodontally healthy, 68 as affected with gingivitis and 57 as affected with periodontitis. The data pertaining to the tongue coating area and tongue coating severity was analysed according to the proposed classification and the following observations were made as seen in Table 2. One way ANOVA f test indicated significant differences between the overall comparison of scores of TCA, TCS and FTCS when all the three periodontal status participants were considered. These parameters exhibited significant differences with Tukey’s post hoc test which was used for pairwise comparison between the healthy when compared with gingivitis and healthy when compared with periodontitis patients. However, comparison of mean values of these parameters in gingivitis and periodontitis patients though they were greater for periodontitis patients did not achieve statistical significance. (Table 3)

**Table 2:** Comparison of tongue coating scores in gender

	Tongue coating area score Mean (SD)	Tongue coating severity score Mean (SD)	Final tongue coating severity score Mean (SD)
Males (*N* = 100)	7.35 (1.97)	4.11 (1.61)	11.47 (2.86)
Females (*N* = 100)	7.16 (2.12)	4.32 (1.68)	11.48 (3.14)
Unpaired t test	t = 0.655	t = −0.901	t = −0.023
P value, Significance	*p* = 0.514 (NS)	*p* = 0.369 (NS)	*P* = 0.981 (NS)

**Table 3:** Comparison of tongue coating scores in different clinical diagnosis

	Tongue coating area score Mean (SD)	Tongue coating severity score Mean (SD)	Final tongue coating severity score Mean (SD)
Healthy (*N* = 75)	6.33 (2.38)	3.5 (1.23)	9.84 (2.85)
Gingivitis (*N* = 68)	7.69 (1.69)	4.32 (1.61)	12.01 (2.57)
Periodontitis (*N* = 57)	7.94 (1.45)	5.01 (1.77)	12.98 (2.5)
One way Anova F test value	F = 13.990	F = 15.931	F = 23.862
p value Significance (overall)	*p* < 0.001**	*p* < 0.001**	*p* < 0.001**
Healthy vs. Gingivitis^	*p* < 0.001**	*p* = 0.005*	*p* < 0.001**
Healthy vs. Periodontitis^	*p* < 0.001**	*p* < 0.001**	*p* < 0.001**
Gingivitis vs. Periodontitis^	*p* = 0.740	*p* = 0.034*	*p* = 0.117

**p < 0.001 – highly statistical significant

^ p value (pairwise) comparison done using Tukey’s post hoc test

The presence of oral malodour was evaluated amongst the study participants which revealed that it was present in 142 patients while it was absent in 58 patients. These participants were then compared for halitosis based on TCA, TCS and FTCS scores using unpaired t test which revealed significant differences. (Table 4)

**Table 4:** Comparison of tongue coating scores with presence/absence of halitosis

	Tongue coating area score Mean (SD)	Tongue coating severity score Mean (SD)	Final tongue coating severity score Mean (SD)
Present (*n* = 142)	8.15 (1.25)	4.61 (1.7)	12.77 (2.08)
Absent (*n* = 58)	5.05 (1.95)	3.24 (0.94)	8.29 (2.49)
Unpaired t test	t = 13.367	t = 5.759	t = 13.015
P value, Significance	*p* < 0.001**	*p* < 0.001 **	*p* < 0.001**

**p < 0.001 – highly statistical significant

When comparing genders, males demonstrated slightly higher mean TCA values, whereas females exhibited higher mean TCS and FTCS values. However, these differences did not reach statistical significance (Table 2). Thus, while variations in mean scores were observed, they should be interpreted as trends rather than definitive differences. Future studies could benefit from reporting medians and ranges, in addition to means, to better capture individual variability in tongue coating severity.

## Discussion

The oral cavity is abundant with microbial presence even in health and which is further increased quantitatively and qualitatively in diseased conditions. These microbial colonies accumulate in various niches within the oral cavity including gingival sulcus, periodontal pockets, retromolar areas, interproximal areas and the dorsum of the tongue [16]. The microbes are densely populated over the dorsum of the tongue and are similar in nature to the other oral microbes which has been previously reported owing to their similarity in terms of microbial composition and pH experiments conducted [6, 17]. Oral malodour has its origin from several of the intra and extraoral sources, but the intraoral conditions predominate amongst them. Reports in the literature have indicated that the malodour is related to the tongue coatings and periodontal status of the individual [18, 19]. In contrast, however not all patients affected with gingivitis and/or periodontitis complain about oral malodour and vice versa. Additionally, the existing tongue coating assessment methods seem to be complicated with a lot of subjectivity in the evaluation. Considering these limitations, a new Tongue Coatings Severity Index is proposed and through this study it was planned to evaluate the prevalence, distribution and severity of tongue coatings according to the proposed classification.

The present study was conducted on equal number of males and females thereby enabling the distinction between gender variations which occur especially in females due to the hormonal changes. Though in one of the previous studies, the authors reported no association between the tongue coatings and hormonal changes it was also mentioned that only half of the female participants were aware of their menstrual phase thereby negating the observation [20]. The results obtained in this study can be related to the set of papillae and taste buds being prominent in females than the males which makes the dorsal surface vulnerable for enhanced accumulation of microbial deposits [21]. This finding of greater papillary density was perceived to be more reliable than some of the previous investigations as it was carried out on a larger population base with equal gender distribution.

The present study is perhaps one of the few studies which has evaluated the association of tongue coatings with the periodontal status of the participants. The findings revealed a positive association indicating that as the periodontal status deteriorated in severity, the TCA, TCS and FTCS scores increased considerably. It is presumed that the dorsal surface of the tongue offers a large surface area as well as a conducive environment for the oral microorganisms to populate and grow along with the accumulation of considerable amounts of desquamated epithelial cells and dead leukocytes. Since the microorganisms in the subgingival environment and the tongue coatings are almost the same, they have a combined effect on periodontal status and oral malodour [22]. 

Previous reports in the literature have evaluated the oral malodour and correlated it with the presence of volatile sulphur compounds, methyl mercaptan/hydrogen sulphide ratios which were found to be elevated in periodontally affected patients along with the increasing severity [23, 24]. The present study evaluated oral malodour based on the TCA, TCS and FTCS scores which indicated a positive association and which was statistically significant. It is estimated that the periodontopathic microorganisms metabolise cysteine and methionine to produce the volatile sulphur compounds which leads to oral malodour [25]. The findings of the present study are similar to the ones reported earlier wherein it was observed that the tongue coating was found to be more in periodontally diseased individuals [9]. The present study can be considered to be more precise as all the three components i.e. TCA, TCS and FTCS scores were evaluated and which confirm this observation. One of the distinctive features in this trial is the significant difference which was observed when all the three parameters of TCA, TCS and FTCS were compared between healthy patients with gingivitis and periodontitis patients.

With regards to the proposed Tongue Coatings Severity Index when compared to the existing indices such as those described by Miyazaki et al. which requires the dorsal surface of the tongue to be divided into nine equal surfaces [12], Winkel et al. which scores the tongue surface in six Sections [26]. and Kim et al. which requires complex digital imaging analysis the present index is simple for clinical use, does not require advanced instruments and can be recorded within a few minutes [27]. Moreover, the proposed index enables the clinician not only to record the coatings on tongue surface area but also the severity of it. Lundgren et al. have mentioned that for reliability of an index the recordings should be reproducible, should possess simplified scoring criteria and minimal recording time [28]. All the above-mentioned aspects are achievable through this index which has been demonstrated in this case with excellent inter examiner reliability, simplified scoring criteria through reduction of the scoring sections and the minimal time required for recording.

It must also be acknowledged that dietary habits, hydration status, and tongue cleaning practices were not recorded and could have influenced the observed outcomes. Furthermore, when compared with existing indices such as Miyazaki’s method, Winkel’s index, or digital imaging systems, the proposed Tongue Coatings Severity Index is simpler, less time-consuming, and more feasible for clinical use.

The clinical applicability of the proposed TCSI lies in its ability to provide a quick, reproducible, and reliable measure of tongue coatings, making it useful in everyday practice. It may serve as a screening tool for halitosis in dental clinics, assist in monitoring disease progression and treatment outcomes in patients with gingivitis or periodontitis, and potentially be applied in community-level surveys for public health purposes. Moreover, beyond its periodontal implications, tongue coating assessment holds promise in systemic health evaluation. Conditions such as diabetes mellitus, gastrointestinal disorders, and respiratory diseases have been reported to manifest changes in tongue coatings, and future studies should explore the utility of TCSI in these contexts. Such applications may expand the relevance of this index well beyond periodontal care, integrating it into broader medical and dental practice.

The current clinical trial has a few limitations. Confounding factors or habits such as tongue cleaning, nature of soft food intake, tea and coffee consumption which are known to influence the tongue coatings to a certain extent were not considered in the evaluation. Also, since the trial was conducted on a limited population generalisation of the results would be a limitation. Future investigations should incorporate blinding of examiners to participants’ periodontal status in order to minimize assessment bias. Additionally, systematic recording of confounding factors such as dietary habits, hydration levels, and oral hygiene practices—including tongue cleaning—would improve the robustness of results. An additional limitation of the present study is that examiners were not blinded to the periodontal status of participants, which could have introduced assessment bias. Future studies should incorporate examiner blinding to strengthen the reliability of findings. Expanding the age range to include elderly individuals, who are particularly prone to thicker coatings due to reduced salivary flow, is also recommended to enhance the generalizability of findings.

## Conclusion

Even though statistical significance was not achieved, the observed trend showed elevated TCS and FTCS values among females, implying a comparatively higher degree of tongue coatings. A positive association was observed between periodontal status and tongue coating scores. The proposed Tongue Coatings Severity Index provides clinicians with a simple and reliable method to evaluate both the extent and severity of coatings within minimal time. We recommend its integration into routine periodontal evaluations and advocate further validation in populations with systemic conditions such as diabetes and viral infections to broaden its clinical applications. The proposed index demonstrates simplicity, reliability, and potential clinical utility. Validation in larger and diverse populations is required before widespread adoption.

## Data Availability

Data is provided within the manuscript.
